# An Assessment of the Knowledge of Oral Isotretinoin (Roaccutane) Treatment Among Pharmacy Students in Saudi Arabia

**DOI:** 10.7759/cureus.40388

**Published:** 2023-06-13

**Authors:** Azizah M Malebari, Hussain T Bakhsh, Renad M Musairi, Jumana O Alghamdi, Albatoul A Alhaddad

**Affiliations:** 1 Department of Pharmaceutical Chemistry, Faculty of Pharmacy, King Abdulaziz University, Jeddah, SAU; 2 Department of Pharmacy Practice, Faculty of Pharmacy, King Abdulaziz University, Jeddah, SAU; 3 Faculty of Pharmacy, King Abdulaziz University, Jeddah, SAU

**Keywords:** saudi arabia, pharmacy students, teratogenicity, roaccutane, oral isotretinoin

## Abstract

Background: Oral isotretinoin (Roaccutane) is one of the most effective treatments for severe acne. However, it displays significant side effects such as teratogenicity and psychological adverse events. Previous studies have reported inadequate awareness of community pharmacists and the general population regarding the medication's potential risks and adverse effects. The aim of this study is to assess pharmacy students’ awareness and knowledge about the appropriate use of oral isotretinoin (known as Roaccutane) and its associated side effects in Saudi Arabia.

Methods: This is a cross-sectional study that uses a validated online questionnaire adopted from the literature distributed among pharmacy students between September 2021 and November 2021.

Results: This study includes 1044 pharmacy students from multiple regions of Saudi Arabia. Among the total number of students included, 47.5% of the participants had used oral isotretinoin before or had a close family member who had used it previously. The most well-known side effect reported is skin dryness (87.7%), followed by teratogenicity (45.2%) and depression (37.9%). Most of the students (90.6%) know that isotretinoin’s use is contraindicated in pregnancy. Despite this, only 39.6% of the participants state that married women of childbearing age using isotretinoin must utilize two types of contraception. There was a significant difference between genders in their knowledge about the side effects of the medication (*P*=0.01), as well as the safety precautions that women of childbearing age should take while taking the medication, as females had better knowledge and understanding of the required measures.

Conclusion: The total awareness level of pharmacy students about the most common side effects of isotretinoin is generally high. However, the students’ knowledge about teratogenicity and depression is inadequate. We recommend paying attention to providing better education on the potential risks and precautionary measures related to the use of this medication, especially for women of childbearing age.

## Introduction

Acne vulgaris is a chronic inflammatory skin condition and is one of the most prominent conditions in the world, affecting people both physically and psychologically [1,2]. Approximately 9.4% of the world’s population is affected by acne, making it the eighth most prevalent disease in the world [3]. The Global Burden of Disease data show that 85% of young adults ranging from 12 to 25 years old are affected by acne [4]. In Saudi Arabia, the prevalence of acne is around 65%, and it has been found that adolescents and young females are more susceptible to it than males [5-7].

Among the prescribed medications for severe refractory nodulocystic acne, including topical retinoids, hormonal therapies, and oral antibiotics, oral isotretinoin (commonly known as Roaccutane) stands out as one of the most effective therapies. It belongs to the class of retinoids, derived from vitamin A, and works by reducing the size of sebaceous glands and decreasing sebum production [8].

Despite the high efficacy of isotretinoin in treating acne, it displays numerous significant side effects, with dry skin and lips being the most commonly reported, affecting 100% of patients [9]. The effect of oral isotretinoin on serum lipids, namely a significant elevation in blood triglyceride and cholesterol levels, was also reported [10]. Additionally, in some case reports, isotretinoin was found to cause pancreatitis secondary to hypertriglyceridemia [11-13]. It also has a risk effect on bone health, causing a reduction in bone mineral density [14]. In addition, the US Food and Drug Administration reported adverse psychiatric events with isotretinoin use, including anxiety and depressive disorder [15]. Other reported adverse effects include irritable bowel syndrome (IBS) and an increased risk of ulcerative colitis, which are associated with higher doses of isotretinoin [16,17].

The most significant adverse effect of isotretinoin treatment is teratogenicity, with an estimated 20-35% risk to infants exposed to the medication. Teratogenicity associated with isotretinoin use includes malformations in the central nervous system, fetal cardiovascular defects, external ear abnormalities, and an increased risk of spontaneous abortion [18,19]. Therefore, the medication isotretinoin is contraindicated in pregnancy and women of childbearing age unless they are participating in a pregnancy prevention program (PPP) [20-24]. Women of childbearing age are educated and assisted in preventing pregnancies during treatment with isotretinoin. As part of the PPP implementation process, informed consent is obtained from patients, they are educated about reliable contraceptive methods, regular pregnancy tests are conducted, and accurate documentation is maintained. Providing ongoing guidance and emphasizing contraceptive measures, PPPs contribute to minimizing the risk of teratogenic effects associated with isotretinoin. Despite these efforts, many studies have reported poor compliance with isotretinoin-related PPPs, especially pertaining to the use of an effective contraceptive method and pregnancy tests during the treatment course, as well as signing a medical consent form [22,25].

Many studies were performed to assess the public’s awareness of isotretinoin use and its adverse effects in different cities in Saudi Arabia [16,22,26-30]. It was found that the majority of people are aware of the side effects of isotretinoin. They also reported that the most common and well-known side effect is dryness of the face and lips. On the other hand, although female patients are adequately aware of isotretinoin’s teratogenicity, they lack awareness of safe practices during the treatment course while at a childbearing age, such as using two types of contraceptives. Moreover, there is still insufficient awareness of the other significant side effects of the medication, such as depression, increased blood cholesterol levels, and liver dysfunction.

A study conducted in 2019 on female Saudi college students studying different specialties showed a variation in knowledge about the side effects of isotretinoin per college major [31]. Furthermore, a recent study conducted atTaibah University, Medina, Saudi Arabia assessed female medical and nursing students’ awareness of oral isotretinoin therapy. It reported that the majority of the students had insufficient knowledge about isotretinoin therapy, requiring that more attention be paid to educational programs to improve their knowledge and level of awareness [32]. In terms of pharmacists’ knowledge, a study conducted in Saudi Arabia showed that pharmacists at community pharmacies are not adequately aware of the proper use and risks of oral isotretinoin [33]. Pharmacists play a crucial role in obtaining patient consent and counseling for oral isotretinoin. They educate about risks, emphasize contraception, and collaborate with medical professionals for safe medication use. Therefore, this study aims to evaluate and assess the general knowledge about oral isotretinoin therapy among pharmacy students at different universities in Saudi Arabia. For this assessment, we include awareness of the side effects of isotretinoin, its teratogenic potential, and the risks and safety precautions related to its use.

## Materials and methods

Study design

It is a cross-sectional study that used an online validated questionnaire to collect data from pharmacy students. The questions in the survey were adapted from previous studies performed in Saudi Arabia [28,30-32]. The pilot questionnaire, administered to expert pharmacists and pharmacy students, assessed the content validity of the study. The study included pharmacy students who were in the advanced stage of their Bachelor of Science in Pharmacy program, specifically those in the fourth and fifth years, as well as those in the sixth year, which corresponds to the internship period of their training. The survey was distributed through social media platforms such as WhatsApp and Twitter.

Study population

The study included pharmacy students from approximately 20 private and public universities in Saudi Arabia, representing a diverse mix of institutions offering pharmacy programs. The study excluded participants who did not complete the questionnaire, pharmacy students from universities outside of Saudi Arabia, and participants in their first, second, and third years of the pharmacy program.

Sampling methodology

The survey was conducted among pharmacy students in Saudi Arabia from September to November 2021. A written consent form was obtained from all participants before they filled out the questionnaire. The questionnaire consisted of three parts and was written in Arabic and English languages. The first part included information about demographic data such as the name of the university, gender, marital status, level year in pharmacy, and academic grade. The second part evaluated pharmacy students’ knowledge about different oral isotretinoin’s adverse effects including skin dryness, teratogenicity, depression, osteoporosis, and increased liver enzymes. The third part evaluated pharmacy students’ knowledge about isotretinoin use instructions such as the necessity to use sunscreen with isotretinoin and the importance of undergoing regular laboratory checks in addition to the safety precaution and potential risks of isotretinoin use during pregnancy, depression, and blood donation.

Data analysis

Data were extracted, reviewed, coded, and entered into SPSS Statistics version 24.0 (IBM Corp. Released 2016. IBM SPSS Statistics for Windows, Version 24.0. Armonk, NY: IBM Corp.). The results were presented as frequencies and percentages. Categorical data were compared using the chi-square test. A significant association was determined by a p-value of <0.05.

Ethical approval

The study was approved by the Biomedical Research Ethics Unit, Faculty of Medicine, King Abdulaziz University (reference No. 21-399).

## Results

This study included responses to the questionnaire from 1044 pharmacy students from different universities in Saudi Arabia. Table 1 shows the demographic characteristics of the students. The majority of the participants were female (60.7%), while males comprised only 39.3% of the sample population. Additionally, most of the participants were single (95.5%), while only 4.2% were married. Participants were categorized into three groups based on their academic year: fourth year (37%), fifth year (34.7%), and sixth year (28.4%). The students were also categorized into four categories based on their academic grades: A (47.6%), B (40.3%), C (11.1%), and D (1%). Among the total number of students included, 47.5% had used oral isotretinoin or had a close family member who had used it previously, while 52.6% had never used the medication.

**Table 1:** Demographic characteristics of the participants (n=1044)

Demographic characteristics of the pharmacy students (n=1044)	N (%)
Gender:	
Female	634 (60.7)
Male	410 (39.3)
Year:	
Fourth year	386 (37)
Fifth year	362 (34.7)
Sixth year	296 (28.4)
Academic grade:	
A	497 (47.6)
B	421 (40.3)
C	116 (11.1)
D	10 (1)
Social status:	
Single	997 (95.5)
Married	44 (4.2)
Other	3 (0.03)
Previous use of the medication by the student or a close family member:	
Yes	495 (47.4)
No	549 (52.6)

Table 2 displays the questions about oral isotretinoin that the pharmacy students had to respond to. The students were allowed to choose more than one answer, with some of the choices being correct and some incorrect. The most commonly known side effect was dryness of the skin, lips, and eyes, with 87.6% of the students responding with this, while 37.8% of the participants mentioned depression. This was followed by spontaneous abortion, osteoporosis, and pancreatitis. All of these responses were correct. In terms of the incorrect responses for side effects, 19.6% of the students selected nausea and vomiting, while 16.6% believed that oral isotretinoin might lead to a sleep disorder. Approximately 77.5% of the students were aware of the effect of oral isotretinoin on the patient’s liver profile, while 52.9% were aware of its effect on the patient’s lipid profile. Regarding the incorrect answers for laboratory monitoring, the percentages of the responses were as follows: kidney profile (36.7%), glucose level (8.8%), and hemoglobin A1C (6.8%). In terms of knowledge about the frequency of laboratory follow-ups, 84.8% of pharmacy students chose “before starting the treatment,” 67.1% chose “during the treatment,” and only 34.3% chose “after the treatment.” Regardless, most of the students (90.6%) knew that oral isotretinoin use is contraindicated during pregnancy. However, only 29.4% of the students had knowledge about the effect of oral isotretinoin on the patient’s mental state, stipulating that the medication’s use is contraindicated in those with depression. In addition, only 10.4% of the students knew that the medication is contraindicated in patients diagnosed with IBS.

**Table 2:** Knowledge and awareness of isotretinoin among pharmacy students IBS: irritable bowel syndrome *Counts and percentages were used to summarize the responses *Students may choose more than one answer to the question

Questions to pharmacy students	Answers	N (%)
Which of the following side effects are caused by long-term use of oral isotretinoin?	Dryness of the skin, lips, eyes, and body (correct side effect)	915 (87.6)
	Depression and suicidal thoughts (correct side effect)	395 (37.8)
	Abortion (correct side effect)	207 (19.8)
	Osteoporosis (correct side effect)	186 (17.8)
	Pancreatitis (correct side effect)	95 (9.1)
	Sleep disorder (incorrect side effect)	173 (16.6)
	Nausea and vomiting (incorrect side effect)	205(19.6)
Which of the following laboratory values may increase during the treatment course of oral isotretinoin?	Liver profile (correct laboratory value)	809 (77.5)
	Lipid profile (correct laboratory value)	552 (52.9)
	Kidney profile (incorrect laboratory value)	383 (36.7)
	Glucose level (incorrect laboratory value)	92 (8.8)
	Hemoglobin A1C (incorrect laboratory value)	71 (6.8)
In the course of oral isotretinoin therapy, when are laboratory tests (full blood count, fasting lipids, liver function test) typically performed?	Before starting the treatment course	885 (84.8)
	During the treatment course	701 (67.1)
	After the treatment course	358 (34.3)
Oral isotretinoin is contraindicated in which of the following cases?	Pregnancy	943 (90.6)
	Depression	306 (29.4)
	IBS	108 (10.4)
	Diabetes	121 (11.6)
	Anemia	127 (12.2)
	Epilepsy	128 (12.3)
Which of the following statements regarding oral isotretinoin use for women of childbearing age are correct?	Oral isotretinoin use is contraindicated in pregnancy because it is teratogenic	784 (75.6)
	Oral isotretinoin must be discontinued at least one month before conception. For both male and female	570 (55)
	Married women of childbearing age using oral isotretinoin must be on two methods of contraception	410 (39.5)
	A pregnancy test is required only before starting the treatment course with oral isotretinoin	273 (26.3)
Which of the following statements regarding treatment with the medication oral isotretinoin are correct?	It’s important to avoid sunlight and use sunscreen while using oral isotretinoin	772 (73.9)
	The treatment course duration of the medication oral isotretinoin is at least six months for effective results	661 (63.3)
	There is a potential for relapse after the treatment course ends	380 (36.4)
	Patients on oral isotretinoin cannot donate blood during the treatment course	310 (29.7)
	Oral isotretinoin might cause weight gain during the treatment course	263 (25.2)
Which of the following patients are required to read and sign the consent form for oral isotretinoin use?	Females are required to read and sign the consent form	1016 (97.3)
	Males are required to read and sign the consent form	444 (42.5)
What are the sources of your information about oral isotretinoin?	Educational material	524 (50.2)
	Friends and family	529 (50.7)
	Social media	377 (36.1)
	Personal use	50 (4.8)
	Physician	24 (2.3)
	Other	39 (3.7)

The student’s knowledge about the precautionary measures that women of childbearing age should take when using oral isotretinoin is shown in Table 2. That is, 55% of the pharmacy students stated that both males and females should discontinue the use of oral isotretinoin at least one month before trying to conceive. On the other hand, only 39.6% stated that married women of childbearing age using oral isotretinoin must be on two types of contraception. Further, 26.3% of the students stated that a pregnancy test is required only before starting a course of oral isotretinoin.

In addition, most of the students (73.9%) were aware of the importance of avoiding sun exposure and using sunscreen while using oral isotretinoin, while only 29.7% were aware that patients on oral isotretinoin cannot donate blood. Finally, around 60% of the pharmacy students had knowledge about the recommended course duration, stating that the treatment course must be at least six months for effective results.

**Figure 1:**
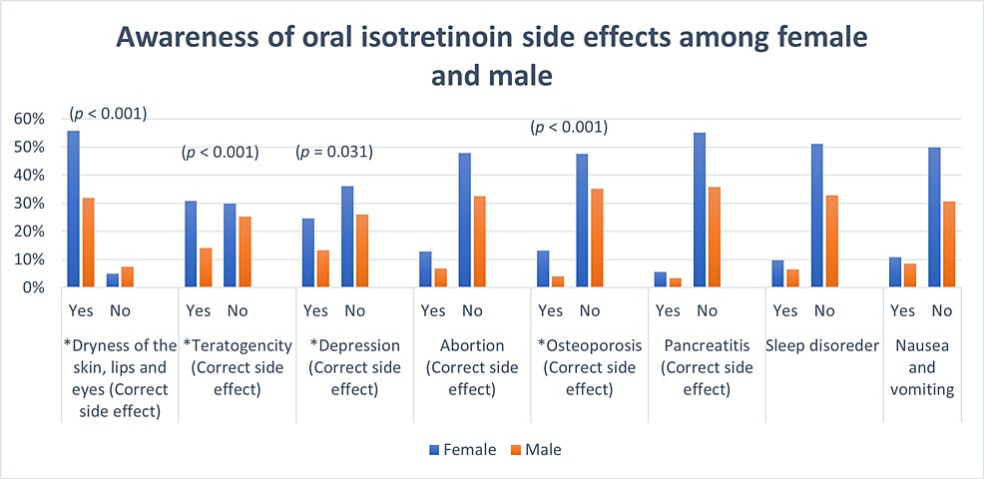
Awareness of isotretinoin side effects among males and females *indicates significant results (*p*-value < 0.05)

**Figure 2:**
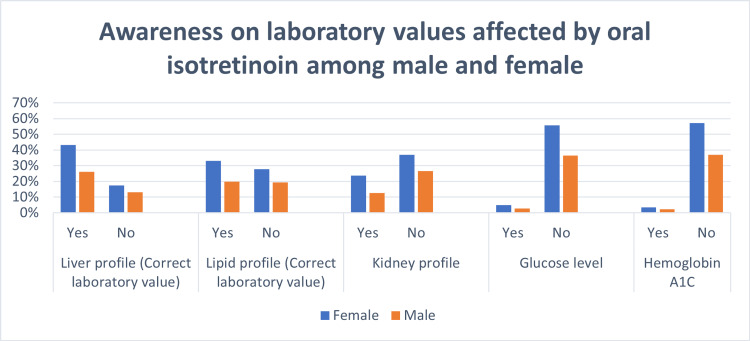
Comparison of the knowledge of laboratory values affected by oral isotretinoin treatment between males and females

The differences between males' and females’ knowledge of the correct side effects of oral isotretinoin are shown in Table 3 and Figure 1. There was a significant difference between genders in knowledge about the correct side effects of teratogenicity (p < 0.001); dryness of the skin, lips, eyes, and body (p < 0.001); and depression (p = 0.03), as females were more aware of the correct side effects. Regarding the incorrect side effects of a sleep disorder, nausea, and vomiting, there were no statistical differences between males and females. For laboratory monitoring, Figure 2 shows that there was no significant difference between males' and females’ knowledge about the importance of monitoring liver and lipid profile values while using oral isotretinoin. In addition, there was no significant difference between the genders in their selection of the incorrect laboratory value monitoring methods, such as the kidney profile, glucose level, and hemoglobin A1C.

**Table 3:** Awareness of isotretinoin side effects among males and females *Counts and percentages were used to summarize the responses *statistical analysis was performed using the chi-square test of independence *The significant p-values are denoted in bold

Side effect	Answer	Female N (%)	Male N (%)	p-value
Dryness of the skin, lips, eyes, and body	Yes	582 (55.7)	333 (31.9)	0.01
	No	52 (5.0)	77 (7.4)	
Depression	Yes	257 (24.6)	139(13.3)	0.03
	No	377 (36.1)	271 (26.0)	
Abortion	Yes	134 (12.8)	71 (6.8)	0.12
	No	500 (47.9)	339 (32.5)	
Osteoporosis	Yes	138 (13.2)	42 (4.0)	0.01
	No	496 (47.5)	368 (35.2)	
Pancreatitis	Yes	58 (5.6)	36 (3.4)	0.83
	No	576 (55.2)	374 (35.8)	
Sleep disorder	Yes	101 (9.7)	68 (6.5)	0.77
	No	533 (51.1)	342 (32.8)	
Nausea and vomiting	Yes	114 (10.9)	89 (8.5)	0.13
	No	520 (49.8)	321 (30.7)	

**Figure 3:**
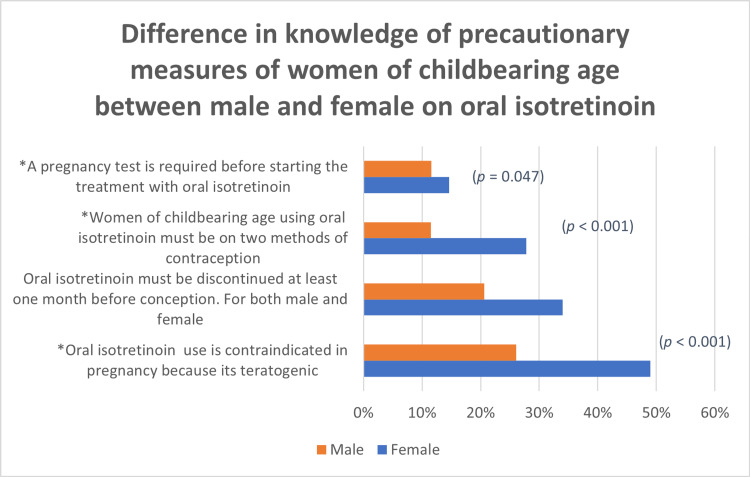
Difference in the knowledge of precautionary measures of women of childbearing age between males and females on oral isotretinoin *indicates significant results (*p*-value < 0.05)

Regarding precautionary measures that women of childbearing age should take when on the medication, there was a significant difference in knowledge between males and females, as females were generally more aware of the necessary precautionary measures to be taken when using the medication. For instance, females more often stated that married women of childbearing age using oral isotretinoin must be on two types of contraception (p < 0.001) and that oral isotretinoin use is contraindicated in pregnancy because it is teratogenic (p < 0.001). However, there was no difference seen between genders in knowing that both males and females should discontinue oral isotretinoin at least one month before trying to conceive (Figure 3).

The differences between users' and non-users’ knowledge about the correct side effects of oral isotretinoin are shown in Table 4. There was no significant difference between users' and non-users’ knowledge of the majority of the correct side effects. Furthermore, around 50% of the pharmacy students suggested two main sources of knowledge about oral isotretinoin: educational material and friends and family, while some of the students also mentioned social media (36.1%), personal use (4.8%), and physicians (2.3%) as sources of information (Table 2).

**Table 4:** Awareness of isotretinoin side effects among users and non-users *Counts and percentages were used to summarize the responses *Statistical analysis was performed using the chi-square test of independence *The significant p-values are denoted in bold

Side effect	Answer	User N (%)	Non-user N (%)	p-value
Dryness of the skin, lips, eyes, and body	Yes	459 (44.0)	456 (43.7)	0.01
	No	90 (8.6)	39 (3.7)	
Depression	Yes	201 (19.3)	195 (18.7)	0.35
	No	348 (33.3)	300 (28.7)	
Abortion	Yes	110 (10.5)	95 (9.1)	0.73
	No	439 (42.0)	400 (38.3)	
Osteoporosis	Yes	85 (8.1)	95 (9.1)	0.11
	No	464 (44.4)	400 (38.3)	
Pancreatitis	Yes	57 (5.5)	37 (3.5)	0.10
	No	492 (47.1)	458 (43.9)	
Sleep disorder	Yes	88 (8.4)	81 (7.8)	0.88
	No	461 (44.2)	414 (39.7)	
Nausea and vomiting	Yes	119 (11.4)	84 (8.0)	0.05
	No	430 (41.2)	411 (39.4)	

## Discussion

Oral isotretinoin is a vitamin A derivative, commercially known as Roaccutane, that has shown the greatest efficacy in treating moderate to severe acne. Due to the increase in the use of oral isotretinoin in Saudi Arabia, ensuring that the population has adequate levels of knowledge about and awareness of the safe use of the medication has become a significant matter. Previous studies have been conducted locally to measure the public’s awareness of and knowledge about using oral isotretinoin, but they revealed a lack of both. Furthermore, during our literature review, we found only two studies conducted among college students. To the best of our knowledge, no similar previous research assessing and measuring the awareness of oral isotretinoin has been conducted among pharmacy students from different universities in Saudi Arabia.

In the present study, we aimed to assess pharmacy students’ knowledge about and awareness of the safety and precautionary measures to be taken when using oral isotretinoin. Out of 1044 pharmacy students, more than half of the respondents were females, and approximately half of the students had previously used oral isotretinoin or had a close family member who had used it. We found that the majority of pharmacy students had a good awareness of the common side effects of the medication, such as dryness of the skin, lips, and eyes, as well as depression and spontaneous abortion. These results agree with those of similar previous studies conducted among nursing and medical students at Taibah University [32]. Our findings also reveal that pharmacy students have poor awareness of depression as a side effect when compared to a previous study conducted among the population in Jeddah [28].

Concerning the laboratory investigations required during oral isotretinoin treatment, it is known that using oral isotretinoin can alter triglyceride AST and ALT levels [34]. A study showed that the differences in both triglyceride and cholesterol levels before and after oral isotretinoin therapy are statistically significant [35]. In our study, more than 50% of the pharmacy students were aware that liver and lipid profiles are laboratory investigations required during a course of isotretinoin. These findings are consistent with those of a previous study on the general population’s awareness of oral isotretinoin use [28]. Regarding the frequency of the laboratory tests while using oral isotretinoin, the majority of pharmacy students were aware of the importance of performing laboratory investigations either before or during the treatment course, while only 34.3% were aware of the necessity of conducting these laboratory investigations after the treatment course. It is well known, however, that these laboratory investigations are crucial and should be done monthly from the beginning of the treatment course [36].

Oral isotretinoin use in women of childbearing age has become a threatening public health issue due to its risks of teratogenic side effects and spontaneous abortions [25]. Many different programs such as the PPP, System to Manage Accutane-Related Teratogenicity (SMART), and iPLEDGE (an obligatory distribution program for oral isotretinoin use in the US) have been established to minimize the birth defects that oral isotretinoin may cause [16,37]. These programs involve the user signing a medical consent form, as well as receiving a contraceptive guide and warnings about oral isotretinoin use. In addition, these programs emphasize using two types of contraception and taking monthly pregnancy tests during the oral isotretinoin course. In agreement with these precautions, the majority of pharmacy students in this study identified pregnancy as being contraindicated to oral isotretinoin use. This result is consistent with that of a previous study conducted on female medical and nursing students at Taibah University and the female population in Makkah province [16,32]. Females were found to have higher levels of awareness of and knowledge about the precautionary measures and safe use of systemic isotretinoin when compared to males. Nevertheless, this study reveals that pharmacy students in Saudi Arabia are, overall, unaware of the precautionary measures that women of childbearing age should take while using oral isotretinoin, such as using proper methods of contraception and performing a pregnancy test before starting the oral isotretinoin course.

In this study, the majority of students (97%) identified the importance of females signing a consent form. However, less than half of the students knew that males are also required to read and sign this medical consent form. Concerning other safety issues and counseling points during oral isotretinoin treatment, around 70% of the students were aware of the importance of avoiding sunlight and using sunscreen during the treatment course, while more than half of the pharmacy students had sufficient knowledge about the most efficacious duration of isotretinoin treatment. On the other hand, the pharmacy students were found to have limited knowledge about not being able to donate blood during the treatment course, as only 29% of the students identified this as a safety precaution. This is in line with what a previous study measuring the awareness of medical and nursing students at Taibah University found [32].

Among pharmacy students who previously used the medication or had a close family member who had used it and non-users, there were no significant differences found in their knowledge about the most common side effects of oral isotretinoin. Contrary to this finding, several Saudi studies have found that previous users of oral isotretinoin have more knowledge about its side effects and safety issues compared to non-users, further showing that the student’s college major (pharmacy or medicine) impacted their knowledge about the side effects of oral isotretinoin [31,32]. While our study found no significant differences in knowledge between previous users and non-users of oral isotretinoin among pharmacy students, it is possible that previous studies involving participants from various backgrounds could yield different results. The variation in findings could be explained by the inclusion of participants from different educational backgrounds. Furthermore, half of our students mentioned educational material and friends and family as their primary sources of information about the side effects of oral isotretinoin [28,30].

The findings of this study revealed that the majority of pharmacy students demonstrated a correct understanding of the contraindication of pregnancy in relation to oral isotretinoin use. However, it is noteworthy that their knowledge regarding other significant side effects such as teratogenicity and the risk of abortion and safety considerations associated with the medication was limited. Moreover, the level of awareness and understanding of the precautionary measures that should be followed by women of childbearing age during isotretinoin treatment was found to be inadequate. This low level of awareness of oral isotretinoin could be related to the lack of education and awareness programs available during their studies. Therefore, these findings indicate the need for devoting more time to educating pharmacy students about the risks and most common side effects of oral isotretinoin, as well as the safety rules and precautions that should be followed during its use. Educating pharmacists on the side effects, precautions, and counseling skills associated with isotretinoin is essential. The development of tailored educational materials and collaboration between healthcare professionals can further enhance patient education. The pharmacist can provide high-quality care to users of oral isotretinoin by emphasizing the pharmacist's role.

Limitations of the study

Despite efforts to ensure accuracy, precision, and representativeness, this study has limitations. Self-reported data may introduce biases and inaccuracies. The study focused on awareness and knowledge, without assessing actual behaviors. Longitudinal data collection was not part of the study design. It is important to consider these limitations when interpreting the findings and to acknowledge the need for further research to provide a more comprehensive understanding of oral isotretinoin use and awareness.

## Conclusions

The awareness of pharmacy students regarding the contraindications associated with oral isotretinoin use during pregnancy is duly acknowledged. However, the primary objective of this study was to evaluate their knowledge pertaining to specific aspects of oral isotretinoin, specifically its teratogenic potential and abortion risk. While most pharmacy students demonstrated awareness of the most common side effects, such as dryness of the skin, lips, and eyes, there was a lack of awareness regarding other serious side effects and safety issues, including teratogenicity and depression. In terms of pregnancy and oral isotretinoin use, the majority of students acknowledged the contraindication. However, their knowledge and awareness of the precautionary measures that women of childbearing age should take during isotretinoin treatment were found to be insufficient. Additionally, there was a notable gender difference, with females exhibiting better knowledge and understanding of the side effects and safety precautions associated with oral isotretinoin. These findings highlight the importance of implementing targeted education and training programs to enhance knowledge and awareness of these crucial precautionary measures among pharmacy students.
